# How the gene ontology evolves

**DOI:** 10.1186/1471-2105-12-325

**Published:** 2011-08-05

**Authors:** Sabina Leonelli, Alexander D Diehl, Karen R Christie, Midori A Harris, Jane Lomax

**Affiliations:** 1 ESRC Centre for Genomics in Society, University of Exeter, St Germans Road, EX4 4PJ Exeter, UK; 2 The Jacobs Neurological Institute, University at Buffalo School of Medicine and Biomedical Sciences, 100 High Street, Buffalo, NY 14203, USA; 3 Department of Genetics, Stanford University, 1501 S. California Ave, Palo Alto, CA 94304-5577, USA; 4 EMBL--European Bioinformatics Institute, Wellcome Trust Genome Campus, Hinxton, Cambridge CB10 1SD, UK

**Keywords:** Gene Ontology, knowledge, maintenance, curation, ontology shifts

## Abstract

**Background:**

Maintaining a bio-ontology in the long term requires improving and updating its contents so that it adequately captures what is known about biological phenomena. This paper illustrates how these processes are carried out, by studying the ways in which curators at the Gene Ontology have hitherto incorporated new knowledge into their resource.

**Results:**

Five types of circumstances are singled out as warranting changes in the ontology: (1) the emergence of anomalies within GO; (2) the extension of the scope of GO; (3) divergence in how terminology is used across user communities; (4) new discoveries that change the meaning of the terms used and their relations to each other; and (5) the extension of the range of relations used to link entities or processes described by GO terms.

**Conclusion:**

This study illustrates the difficulties involved in applying general standards to the development of a specific ontology. Ontology curation aims to produce a faithful representation of knowledge domains as they keep developing, which requires the translation of general guidelines into specific representations of reality and an understanding of how scientific knowledge is produced and constantly updated. In this context, it is important that trained curators with technical expertise in the scientific field(s) in question are involved in supervising ontology shifts and identifying inaccuracies.

## Background

### The Importance of Shifting Ontology

The Gene Ontology [GO] provides a representation of biological knowledge through the use of precisely defined, interrelated terms [1,2]. For GO to successfully underpin data-driven discovery and database searches, the definitions of the terms used, and their relations to each other, need to accurately portray existing biological knowledge about entities and processes. Thus, a key challenge for the long-term maintenance of GO consists of updating its contents to reflect new scientific developments that challenge established biological knowledge [3]. GO curators have been aware of this since the creation of GO [4] and have sought to establish mechanisms of feedback, so that users of GO could alert curators to any discrepancy between the understanding of given entities or processes routinely used within their own fields and the representation of that knowledge provided in the ontology [5]. Indeed, the capability of bio-ontologies such as GO to reflect new developments as they arise has been highlighted as key to their increasing popularity [6,7].

GO was created in 1999 and is thus one of the longest-running ontologies within the Open Biomedical Ontologies (OBO). This paper explores how, in the course of its existence, GO has evolved to represent biological knowledge. We review the changes that have been applied to how GO terms are defined and related to each other, with a view to clarifying whether and how the content of GO has been modified to adequately fit new evidence. Reviewing how GO has been developed over the years is an important way for the users of GO and biologists in general to understand the processes involved in ontology development. This paper aims to provide biologists who are not normally involved in the development of ontologies with an understanding of the amount of conceptual and practical effort needed in this area, as well as the expertise involved. We therefore do not focus on changes implemented to improve the interoperability and internal coherence of GO, e.g. [8]; nor do we discuss how conformance to OBO rules or Basic Formal Ontology (BFO) standards have affected the content of GO, which has been dealt with elsewhere [6]. Our study focuses on cases of *ontology shifts *that occurred in order to improve the ways in which GO represents biological knowledge. Ontology shifts are defined as changes to the biological content of GO, including changes in the definitions of GO terms, the order in which GO terms are situated in the network hierarchy, the relations used to link these terms and the links made between terms and data and/or meta-data.

## Results

### Ontology shift 1: Dealing with anomalies

One type of ontology shift occurs when curators become aware of a mismatch between GO representation and reality, leading to a term being incorrectly related to other terms in the ontology. The discovery of such anomalies leads to revisions of the ontology, which needs to be both internally consistent and faithfully representing reality.

**Figure 1:**
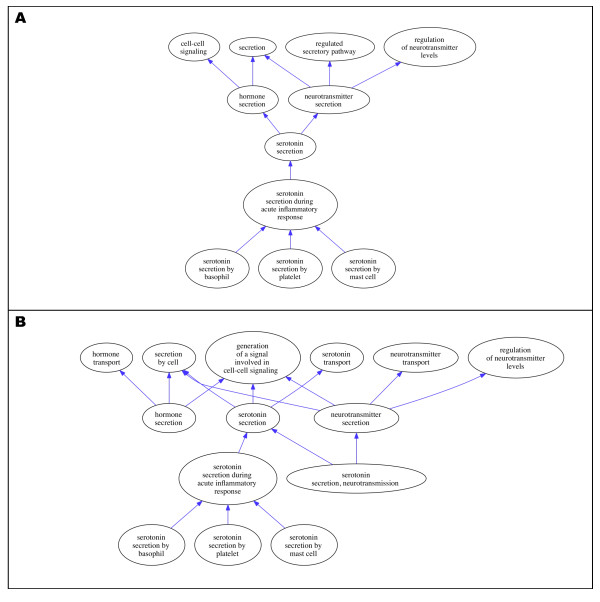
**A shows the initial anomalous placement of the term "serotonin secretion," and B shows its corrected position in the current GO biological process ontology hierarchy**.

As an example, consider the GO process term "serotonin secretion". Serotonin is a small molecule produced by a variety of cells, including neurons, enterochromaffin cells, basophils, and mast cells. It plays several roles in the body, most famously as a neurotransmitter, but also in contraction of the gut and mediation of allergic inflammation by mast cells. The term "serotonin secretion" was initially added to the ontology as an *is_a *descendant of both "hormone secretion" and "neurotransmitter secretion," since the neurotransmitter and hormone roles of this molecule were the only roles considered when the term was developed. When a new term "serotonin secretion during acute inflammatory response" was subsequently created as an *is_a *descendant of "serotonin secretion" (Figure 1A) it was realised that it was not biologically accurate to state that "serotonin secretion during acute inflammatory response" was a subtype of "neurotransmitter secretion," because in inflammatory responses serotonin acts on target cells other than neurons. The erroneous placement of "serotonin secretion" was corrected resulting in the graph shown in Figure 1B. The new term "serotonin secretion, neurotransmission" was added as a subclass of "serotonin secretion" to capture the process of serotonin secretion during neurotransmission.

Another example is the disambiguation of immune responses from defense responses. Regulatory immune responses such as those involved in tolerance induction to non-self antigens, which prevent inappropriate responses to substances in food, for instance, challenge the idea that every "immune response" is also a "defense response". Yet, these two terms were initially represented as synonymous within GO. This situation was discussed during a GO meeting at The Institute for Genomic Research in November 2005. Several biological cases were presented as anomalous under the current description, and it was determined that this warranted a broad shift in the ontology itself. "Immune response" and "defense response" thus became terms that share a common ancestor term but have no direct relationship between them. This enabled curators to distinguish between the two types of responses, while signalling their common origin as a reaction to a stimulus.

### Ontology shift 2: Expanding scope

A second type of ontology shift occurs when GO needs to be extended to cover terminology and data coming from new *research fields, biological issues *or *species*.

The problems caused by the addition of immunological terms illustrate what happens when including knowledge from a new *field *into the ontology. The scope of what GO considered an "immune response" was expanded by a major revision discussed at a GO meeting in 2005 [9] and completed in September 2006 [10], as still it became clear that the concept of having an "immune system" was not restricted to vertebrates. Biologists working in both invertebrate and plant systems used the term "immune system" to describe the cells and biological processes mediating innate immune responses in these organisms [11]. Until the revision, GO had considered immune responses in higher vertebrates only, and terms related to innate immune responses in other organisms, such as the "incompatible interaction" of plants and "melanization defense response" in insects, were found in other areas of GO. After the revision, all the various types of immune responses in different organisms were grouped together as types of "immune response".

What happens when GO is expanded to cover a new *biological issue *is illustrated by the 2004 development of ontology to describe host-parasite interactions. The early versions of GO contained few terms to describe the interactions that occurred between hosts and their symbionts. Those that did exist, such as "evasion of host defense response" and "cell invasion," shared no common ancestor and were often ill-defined with respect to which organism in a particular interaction the term was referring to. For example, the process of cell lysis can be induced in a host organism by its parasite, or can be an endogenous process whereby the immune system destroys its own infected cell, but both were represented by a single term, "cytolysis". In 2004 the PAMGO (Plant-Associated Microbe Gene Ontology, http://pamgo.vbi.vt.edu) Consortium was formed and worked with GO to develop terms relating to host-parasite interactions, specifically for plant parasites. The initial set of around 450 terms added to biological process had a single ancestor term "interaction between organisms" (later to be renamed "multi-organism process") which not only encompassed host-parasite interactions but also processes as diverse as "female pregnancy" and "biofilm formation". The multi-organism process sub-hierarchy now contains over 1300 terms, and in addition there are around 80 terms in cellular component to describe locations within other organisms, such as "host". The introduction of these terms also required a change to the annotation methodology such that information about the taxon of both the species involved in an interaction could be captured (see http://www.geneontology.org/GO.annotation.conventions.shtml#interactions).

**Figure 2:**
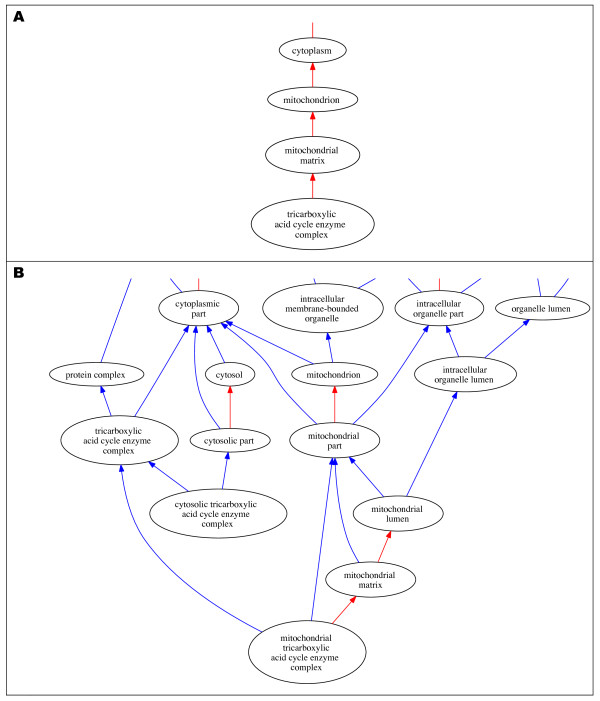
**A shows the initial anomalous parentage of the term "*'tricarboxylic acid cycle enzyme complex*" and B shows the corrected ontology structure in the current GO cellular component ontology hierarchy**.

Finally, ontology shifts have frequently occurred when GO was expanded to include data from a new *species*. GO aims to support the comparative analysis of gene products across species, and its terms need to accommodate differences in the biology of organisms ranging from fruitflies to mice and plants [12]. Especially when grouping together species coming from different kingdoms, GO has been radically modified to avert the danger of biological inaccuracies. When GO was first applied to prokaryotic gene products, for instance, many relationships within the cellular component ontology, and some in the biological process ontology, had to be altered to allow for the fact that prokaryotes do not have nuclei or several other membrane-bounded organelles. For example, the enzyme complexes that carry out the reactions of the TCA cycle (also known as the Krebs cycle or citric acid cycle) are located in the mitochondrial matrix in eukaryotes. Cellular component terms representing these complexes were originally grouped under 'tricarboxylic acid cycle enzyme complex', which was in turn *part_of *"mitochondrial matrix". In bacteria, which do not have mitochondria, analogous complexes are located in the cytosol; the way in which GO related the TCA cycle complex terms to "mitochondrial matrix" was thus inaccurate (Figure 2a). To address this, the existing term was renamed to add "mitochondrial", thus making information that was implicit in the ontology structure explicit in term names. Two new terms were then added, one of which uses the non-location-specific name, and is a descendant of "cytoplasm"; the second new term has a name and path specifying that the complex is located in the cytosol (Figure 2b).

### Ontology shift 3: Dealing with diverging definitions across communities

A third type of ontology shift results from the need to deal with diverging definitions across research communities. Often, given the diversity and fragmentation typical of biological and biomedical research, the same phrase is used in different ways depending on the research context [3]. Maintaining univocity (a word or phrase having a single meaning) is essential in developing unambiguous ontologies, so it is necessary to alter the structure of GO where cases of multiple meaning for a word or phrase arise. Not surprisingly, this type of ontology shift often coincides with ontology shifts of type three detailed above: divergence in the use of terms across communities are commonly discovered when new terminologies, fields or species are added to GO.

The 2001 transition to include plants in the ontology is a case in point. Early on, GO had only one term for gamete formation: "gametogenesis", which was defined as the "generation, maintenance, and proliferation of gametes". The meaning of "gamete" was not specified, but the term and definition were generated with animal gamete formation in mind. Plant biologists, however, use "gametogenesis" to refer to the generation of a gametophyte, that is, a plant in the haploid phase that can produce gametes. An extensive set of changes was required to remove ambiguity in the usage of "gametogenesis", and add terms to represent plant biology. The definition of "gametogenesis" was altered to define gamete as "a haploid reproductive cell" and to remove the mention of proliferation. For plant processes, a new term "gametophyte development" was added, its name and definition clearly referring to the relevant phase of a plant life cycle.

### Ontology Shift 4: Mirroring scientific advance

A fourth type of ontology shift occurs in response to new evidence which changes the understanding of a given entity or process so that its definition and relations to other terms also need to change.

An example of this involves the term "cytoskeleton". For several decades, cytoskeletal structures such as microfilaments, microtubules, and intermediate filaments were observed only in eukaryotic cells, and were therefore thought to be absent from prokaryotic cells. Accordingly, the definition for the GO cellular component term "cytoskeleton" followed one of many dictionary and textbook definitions, beginning "Any of the various filamentous elements that form the internal framework of eukaryotic cells". In recent years, however, evidence has accumulated that bacterial cells do contain cytoskeletal structures [13,14]. To accommodate these discoveries, and to facilitate annotation of bacterial cytoskeletal gene products the GO definition had to be broadened to remove the word "eukaryotic", and with it the restriction on species to which the term could be applied. Deletion of a single word from a term definition thus allowed GO to capture a revolutionary advance in the research community's understanding of both prokaryotic cell organization and the taxonomic distribution of cytoskeletal structures.

The definition of the term "conoid" is another example of how ontology can shift in response to scientific developments. The conoid is a cytoskeletal element that forms part of the apical complex, a distinctive and elaborate structure found in apicomplexan parasites [15]. Based on electron microscopic analysis, the conoid was known to consist of fibers; based on the prevailing hypothesis that the fibers were microtubules, GO included a cellular component term, "conoid", which was an is_a descendant of "microtubule". The definition was: "Coiled microtubules within both the polar and basal rings of the apical complex of an apicomplexan parasite." More recently, Hu et al. [16] showed that the conoid is indeed composed primarily of tubulin, but the tubulin structure differs markedly from that of typical microtubules. This improved understanding of conoid structure had two consequences for "conoid". First, the *is_a *relationship to "microtubule" was removed, because the conoid could no longer be considered a type of microtubule. Second, the text definition was changed to remove information now known to be false and to more accurately describe the structure of a conoid.

### Ontology shift 5: Adding relations

One last type of ontology shift concerns changes to the type of relations deemed to hold between ontology terms. This shift is more complex than the previous four, since it potentially affects the whole ontology and requires a revision of the whole system in order to be implemented.

As originally used in GO, the *part_of *relationship was not rigorously defined. This led to a number of problems, one of the biggest ones being that the *part_of *relationship in GO was being used with different levels of stringency: in some cases *all *of the subclass is part of *some *of the superclass (*all-some*), while in others only *some *of the subclass is part of *some *of the superclass [17]. For example, the TRAMP complex is found only in the nucleus, while the exosome complex is found in both the cytoplasm and the nucleus yet in GO both complexes had the same *part_of *relation to nucleus, with the exosome complex having a further *part_of *relation to cytoplasm. To remedy this situation, the scope of the *part_of *relationship was limited to specifically refer to the *all-some *relationship, and the graph altered accordingly. Further, new relationship types have been introduced [18] to address other consistency issues with the use of part_of in GO:

#### • Regulation Relationships

Before the introduction of the regulation relationships, all regulatory processes in GO were made *part_of *the processes they regulated, which was insufficient to capture the biology because not all regulatory processes are integral to the processes they regulate. For example, a kinase which phosphorylates a transcription factor and thus regulates its translocation from the cytoplasm to the nucleus regulates transcription. However, the kinase is not part of the transcription machinery and thus does not itself play a direct role in the process of transcription.

#### • Has_part relationship

The use of the *part_of *relationship in the spliceosomal component terms led to a logical flaw, sometimes referred to as a true path violation, in the ontology. The term "U5 snRNP" was a *part_of *descendant of both the term "major (U2-dependent) spliceosome" and the term "minor (U12-dependent) spliceosome". Most eukaryotic organisms have two forms of spliceosomes, each of which contains five snRNP complexes, four which are unique to that type, and one which is found in both types of spliceosomes. While it is true biologically that both the major (U2) and the minor (U12) forms of the spliceosome contain the U5 snRNP, any specific U5 snRNP complex is not present in both forms of the spliceosome at the same time. Further, some organisms, e.g. *S. cerevisiae*, do not have the minor spliceosome. The "U5 snRNP" *part_of *the "minor (U12-dependent) spliceosome" relationship leads to the conclusion that the *S. cerevisiae *genes annotated to the term "U5 snRNP" are present in the minor spliceosome, which is not true. During a major revision of the spliceosomal complex terms, new terms were added to represent the different spliceosomal complexes that are recognized during various stages of the spliceosomal assembly/disassembly cycle. The *has_part *relationship was then used to capture the biological relationships between some of the large spliceosomal complexes and the smaller snRNP complexes, thus more accurately describing relationships between complexes and subcomplexes in the cellular component ontology (and similarly in the biological process ontology) that were either misrepresented or not represented previously.

## Discussion

Like other ontologies in OBO, GO can be used as a platform for data sharing only insofar as it accurately captures current biological knowledge. GO terms are expected to refer to real biological entities and processes, and thus the definitions and relations used to characterise these terms need to reflect established knowledge about those entities and processes. OBO view this as a crucial principle underlying the development and use of ontologies in biology, and yet its application in practice is not straightforward. As illustrated by the cases discussed above, ontology shifts tend to happen for a variety of different reasons and to affect the ontology to varying degrees. Some shifts, such as the introduction of regulation and *has_part *relationships in GO (ontology shift 5), have a deep impact on the whole structure of the ontology; other shifts, such as the change in the GO terms related to serotonin secretion (ontology shift 1), have a more limited impact, affecting only a specific part of the ontology. Interestingly, in cases such as the term 'immune response' (ontology shift 3), a small shift in a definition or the choice of a term affects several of the related terms within the ontology. In all of these cases, the expert judgement and manual intervention by curators appears key to the appropriate development of an ontology. This is because carrying out these ontology shifts involves a complex set of skills. To effect the shifts illustrated above, curators are required to find and interpret new information coming from biological research to work out whether, and how, that affects the existing structure and content of GO; and to assess the representation of biological reality given within the GO, so as to judge how, if at all, it needs to be changed to accommodate new information. This means that in order to update GO, its curators need to be able to adequately understand and represent research carried out in contemporary experimental biology. As in the example of the cytoskeleton (ontology shift 4), they need to be able to spot discoveries relevant to how ontology terms are structured and defined, and work out how an ontology needs to change in response to new knowledge. On the one hand, this requires appropriate training in information technology and computer science, so as to be able to design, develop and modify ontologies. On the other hand, curators also need to be familiar with the scientific knowledge that their ontology aims to capture, the methods through which such knowledge is obtained, and the importance attached to specific discoveries and evidence by the relevant research communities.

## Conclusion

The difficult judgments and decision-making processes involved in keeping an ontology up to date explain the relatively slow pace in implementing even well-circumscribed changes. This situation will certainly improve as ontology development becomes increasingly professionalised and automated. The OBO Foundry, for instance, is seeking to identify standard solutions that will hopefully enable the automation of at least some curatorial tasks [6]. Nevertheless, the examples above point to the difficulties involved in applying general standards to the development of a specific ontology. Ontology curation aims to produce a faithful representation of knowledge domains as they keep developing, which requires the translation of general guidelines into specific representations of reality and an understanding of how scientific knowledge is produced and constantly updated. In this context, it is important that trained curators with technical expertise in the scientific field(s) in question are involved in supervising ontology shifts and identifying inaccuracies.

## Methods

This is a qualitative study based on an in-depth analysis of specific examples of ontology shifts, each of which is discussed by the curator responsible for implementing it. The curators involved in this study were recruited by Sabina Leonelli via an email request for collaboration on exploring ontology shifts in GO, to which they freely responded by contributing what they saw as particularly interesting examples from their work. The selection of the examples used here is thus dependent on the specific expertise and interests of the authors of this paper, as usual in qualitative studies of this kind. The examples of ontology shifts are grouped into five categories, depending on the scientific circumstances that warranted them: (1) the emergence of anomalies; (2) the extension of the scope of GO; (3) divergence in how terminology is used across user communities; (4) new discoveries that change the meaning of the terms used and their relations to each other; and (5) the extension of the range of relations used to link entities or processes described by GO terms. Curators selected these specific cases on the basis of what they perceived as their typicality (how well they exemplify other cases within the same category); their intelligibility (the ease with which they could be explained within the word limits of this review); and their significance (the extent to which they impacted the structure and content of GO). A quantitative study would be needed to determine the frequency with which each type of ontology shift has appeared in GO (or any other bio-ontology). Due to the current lack of such quantitative data, there is no relation between the order in which the ontology shifts are listed in this paper and the frequency with which they occur in practice.

## Authors' contributions

SL conceived of the study and its design, co-ordinated all the contributions and drafted the background, discussion and conclusion sections. AD drafted the section on immune responses and serotonin (ontology shift 1), and provided the figures. KC drafted the section on regulation (ontology shift 5). MH contributed the case studies on cytoskeleton, TCA cycle complexes and gametogenesis (ontology shifts 2, 3 and 4). JL participated in the co-ordination and revision of the study, and contributed the case studies on host-parasite interactions and the term conoid (ontology shifts 2 and 4). All authors read and approved the final manuscript.
